# Immunohistochemical and Physiological Research on Farm Animals

**DOI:** 10.3390/ani13040739

**Published:** 2023-02-18

**Authors:** Paola Scocco, Elena De Felice, Alessandro Malfatti

**Affiliations:** School of Biosciences and Veterinary Medicine, University of Camerino (MC), 62032 Camerino, Italy

This Special Issue “Immunohistochemical and Physiological Research on Farm Animals” is dedicated to the application of immunohistochemical and physiological studies carried out on farm animals, including traditional (pig, cow, buffalo, horse, sheep, goat, rabbit, turkey and trout) and emerging farm species (yak, sea bass and zebrafish).

Farm species play very important economic and sociocultural roles, such as food supply, source of income, asset saving, source of employment, soil fertility, livelihood, transport, agricultural traction, agricultural diversification and sustainable agricultural production.

For this reason, it is essential to study the anatomy and the physiology of these species in more detail, in order to improve the knowledge about their morpho-physiological features and to guarantee them a high level of welfare and, in turn, better conditions for farmers, including farm income.

In the last few decades, there has been an exponential increase in publications on immunohistochemistry (IHC) and immunocytochemistry techniques, reflecting the current position that IHC holds in most pathological laboratories.

This Special Issue attempts to demonstrate the relevance of immunohistochemical analysis and its relationship with functions in farm animals. However, taking into account the fact that different methods (such as real-time q-PCR, Western blot, FISH, etc.) are necessary to draw more reliable conclusions, the use of these methods is also considered.

In addition to base research contributions, we asked authors for research with potential applied-purpose studies whose results could be useful in improving farm management practices and the responsible use of natural resources, enhancing the nutraceutical properties of animal-derived products and promoting the circular economy and, in turn, the sustainability of livestock.

A total of 20 papers have been contributed for this Special Issue by 118 authors from four countries, comprising 18 research articles and 2 communications. 

The work of Premi and colleagues [1] investigates the physiological variations affecting plasma analyte concentrations during the pivotal stages of the lactation cycle in healthy multiparous Holstein dairy cows. They analyzed 34 different analytes, including markers of energy metabolism, protein metabolism and kidney function, mineral metabolism, liver function, inflammation and acute phase proteins, and oxidant status, in four different periods: the dry, the postpartum, the early and the late lactation phases. This study provides a guideline of physiological trends affecting plasma analyte concentrations during the different stages of the lactation cycle.

Barile and coworkers [2] identify the use of pregnancy-associated glycoproteins (PAGs) as the best strategy to diagnose pregnancy failures in buffalo (*Bubalus bubalis*). PAGs constitute a large family of glycoproteins expressed in the outer epithelial layer (chorion/trophectoderm) of the placenta in eutherian species. Through a radioimmunoassay applied on blood samples, PAGs were recognized as the best marker for predicting embryonic mortality between 25 and 40 days of gestation in buffalo.

De Felice and colleagues [3] evaluate the effect of a diet supplemented with a standardized powder extract, red (blood) orange and lemon extract (RLE), rich in flavanones, anthocyanins and other polyphenols, on the neuropeptide Y (NPY) distribution in the gastro–entero–pancreatic system of goat (*Capra hircus*) kids. Through single and double immunostaining for the first time, the NPY distribution in the abomasum, duodenum, and pancreas and the co-localization with serotonin (5-HT) were documented. Upon RLE feed supplementation, NPY immunoreactive cells increased significantly in abomasal epithelium and pancreatic islets. NPY is known to regulate gastric acid secretion while on the endocrine pancreas to suppress insulin via autocrine and/or paracrine mechanisms, and to stimulate glucagon secretion. These observations represent a baseline for future studies on the interaction between neuropeptides and polyphenols used as feed additive instead of antibiotics.

Wang and coworkers [4] examine the expression of Mic60 and OPA1, two mitochondrial inner membrane proteins, and the morphology of mitochondria in the myocardium of adult and aged Tibetan sheep. With an integrated approach, including real-time q-PCR, ELISA, immunohistochemistry and the ultrastructure morphology of mitochondria with transmission electron microscopy, the authors suggest that the expression of Mic60 and OPA1 genes and OPA1 protein will reduce, while reducing the capacity of myocardial mitochondria with age.

Melotti and coworkers [5] describe the application of a collagen-based skin-like scaffold (CBSS), manufactured with collagen extracted from sea urchin food waste, to treat experimental skin wounds in sheep (*Ovis aries*). Collagen fibrils in their native conformation with endogen fibril-associated glycosaminoglycans can be extracted from sea urchins, obtaining a biomaterial resembling the in vivo structural microenvironment. Clinical, histopathological, immunohistochemical, and molecular experiments assess CBSS’ effects on the wound healing process. The authors demonstrate the efficacy of this pioneering skin substitute in an in vivo model. The skin substitute supported and stimulated wound healing throughout the whole process; it controlled inflammation, promoted the deposition and maturation of granulation tissue, enhanced re-epithelialization, and induced the formation of skin appendages. This application explores the possibility of deriving high-value and innovative products for veterinary innovative applications from waste materials.

De Carolis and colleagues [6] study ovine pregnancy-associated glycoprotein (oPAG) levels in the plasma of Sarda and Lacaune ewes throughout gestation and in the first month postpartum, using two heterologous radioimmunoassays (RIA-706 and RIA-srPool). For both breeds, these RIA systems were capable of distinguishing pregnant from non-pregnant ewes starting from day 18 of gestation. The diagnosis of pregnancy at early gestation is fundamental to minimize the costs of unproductive animals.

Barbato and coworkers [7] evaluate the effects of cereal supplementation on body condition score and metabolic hormone profile in milking ewes grazing on semi-natural pastures of the Central Apennines in Italy during the grazing summer period. Sheep are the most-bred species in the Central Apennines, where the natural pastures are used as a trophic resource and grazing activity is fundamental to maintain the grassland biodiversity. Increasing summer aridity decreases the grassland pastoral value, negatively affecting animal morpho-functional features and production with detrimental effects on the sustainability of extensive sheep farming. This work represents a part of a wider study aimed at buffering the negative effects of increasing summer drought stress on farm income and maintaining the grassland biodiversity. Through enzyme immunoassays, radioimmunoassay and ELISA on blood samples, Nesfatin-1, insulin, glucagon, leptin, 3-3′-5-triiodothyronine and cortisol were evaluated. The results of this work indicate that nutritional supplementation has protected ewes from the usual lowering of the body condition linked to lactation, and provides a good maintenance of milk production, also determining a better overall body and metabolic state of the animals, which is important at the beginning of the sexual season.

Fan and colleagues [8], with a multidisciplinary approach including immunohistochemistry, Western blot and real-time q-PCR, investigate the expression and distribution of extracellular signal-regulated kinases1/2 (ERK1/2) in the main reproductive organs of female yak (*Bos grunniens*) during different stages. ERKs are an important subfamily of mitogen-activated protein kinases (MAPKs), which regulate various cellular activities and physiological processes. For example, ERK1/2 has a pivotal role in the ovulatory process or in the mechanism of regression and functional maintenance of corpus luteum. In female yak, the expression of ERK1 and ERK2 proteins and mRNA was most pronounced in the ovary in the luteal phase and gestation period, the oviduct in the luteal phase, and the uterus in the gestation period. The histological appearance and physiological process of the main reproductive organs also varied with the different reproductive stages. These results imply that ERK1/2 plays an important role in the regulation of reproductive functions in different physiological situations.

Dall’Aglio and colleagues [9] test the effects of different feed physical forms (different grinding intensities and compactions of the same diet) on the mandibular gland (MG) of growing pigs (*Sus scrofa domesticus*). Samples were analyzed using conventional histochemistry to identify the glycohistochemical profile and using immunohistochemistry to localize aquaporin 5, apelin and apelin receptor. This study demonstrates that different feed physical forms are capable of inducing morphological and functional modifications of the MG. The intense chewing activity related to the highest feed compaction and hardness promotes an increase in pig MG secretion, and saliva becomes more fluid and richer in acid glycoconjugates in order to better lubricate the bolus and protect the mouth mucosae. It has been hypothesized that the apelinergic system is likely involved in the above modifications, enhancing both the fluidity and the quantity of serous saliva.

Palus and coworkers [10] demonstrate the changes in the population of enteric neurons in the porcine stomach in response to the supplementation of low and high acrylamide doses. Using the double immunofluorescence staining method, it was established that supplementation with both doses resulted in an increased number of the cocaine- and amphetamine-regulated transcript (CART), vesicular acetylcholine transporter (VAChT), and neuronal isoform of nitric oxide synthase (nNOS) immunoreactive neurons. The detected alterations of the porcine stomach neuron phenotype suggest an important role of the enteric nervous system in protecting the gastrointestinal tract during acrylamide intoxication.

Toschi and colleagues [11] immunohistochemically analyze the localization in the myenteric plexus of the porcine ileum of both the cannabinoid receptors, namely CB1R and CB2R, the cannabinoid-related receptors TRP vanilloid 1 (TRPV1) and TRP ankyrin 1 (TRPA1), and 5-HT1, a serotonin receptor (5-HT1aR). In the gastro-intestinal tract, cannabinoid receptors are known to regulate motility, secretion, emesis, food intake, and inflammation. The morphological findings of this article could represent a relevant anatomical basis for future functional, pre-clinical and clinical studies assessing the effects of cannabinoids in the management of the hypermotility associated with gastrointestinal inflammatory diseases and pain in pigs.

Tian and coworkers [12] determine the effect of excessive back fat of sows on placental oxidative stress, ATP generation, mitochondrial alterations in content and structure, and mitochondrial function in isolated trophoblasts. Excessive back fat of sows was associated with increased plasma lipid and leptin levels, which were associated with increased systemic (elevated plasma H2O2 level) and placenta (high levels of placental protein carbonylation and GSSG) oxidative stress, likely because of an increase in placental reactive oxygen species (ROS) production and a reduction in antioxidant defenses. In an in vitro model of pig trophoblast cell culture, cytotrophoblasts from the placenta of sows with excessive back fat reveal the decreased mitochondrial maximum respiration and spare respiratory capacity. The data collected show that excessive back fat exacerbates mitochondrial injury induced by increased oxidative stress in pig term placenta, which may have deleterious repercussions on placental activity and, therefore, impair fetal growth and development. 

Jana and Calka [13] analyze the influence of the *E. coli*-induced inflammatory state of the uterus on the neurochemical characteristics of the gilt (crossbred Large White × Landrace pigs) caudal mesenteric ganglion (CaMG) uterus-supplying neurons. After intrauterine bacterial injection, the population of uterine neurons presenting positive staining for dopamine-β-hydroxylase (an enzyme participating in noradrenaline synthesis) and negative staining for galanin, as well as the population of uterine neurons presenting negative staining for dopamine-β-hydroxylase but positive staining for neuropeptide Y, were decreased. Uterine inflammation causes changes in the spatial and neurochemical organization patterns of the CaMG neurons innervating the uterus.

Armando and coworkers [14] examine the so-called epithelial to mesenchymal transition in horse (*Equus caballus*) in a squamous cell carcinoma (SCC) using immunohistochemistry for the first time, thus illustrating an example of tumor cell adaptation during the metastatic process.

Abdel-Wareth and Metwally [15] evaluate the potential effects of thyme essential oil (TEO) as an alternative to dietary antibiotics on the productive performance and serum metabolic profile of male rabbits (*Oryctolagus cuniculus*). TEO levels up to 180 mg/kg can play a major role in improving the productive performance, semen quality, testosterone levels, and the kidney and liver functions, analyzed through blood biochemical assay. 

Preziuso [16], in her communications, investigates the interaction between severe acute respiratory syndrome coronavirus 2 (SARS-CoV-2) spikes and ACE2 of lagomorphs, rabbit, and American pika (*Ochotona princeps*), by means of sequence analysis and structure stimulation. Preziuso proved that the simulated complexes ACE2-SARS-CoV-2-RBD showed a high affinity of ACE2 of lagomorphs to the viral spike protein, suggesting that lagomorphs could be susceptible to SARS-CoV-2.

Prusik and Lewczuk [17] typify the diurnal rhythm of plasma melatonin (MLT) concentration and its regulation by light and endogenous oscillators in turkeys (*Meleagris gallopavo domesticus*). Considering the significance of the domestic turkey as a meat-producing animal and the regulation of MLT of feed intake, plasma analysis showed that MLT was secreted in a daily rhythm with a very high amplitude, but also responds quickly and precisely to changes in light conditions.

With regard to teleost fish, Carminato and coworkers [18] investigated the effects of two different diets (organic vs. conventional) on European sea bass (*Dicentrarchus labrax*) in terms of growing performance, oxidative stress, and contaminant markers. The results of this study, which can be considered a pilot, point out a positive trend in the growing performance of both groups but a greater productivity of conventional fish feed compared to the organic ones on one hand, and significant differences among groups in terms of the oxidative stress and contaminant markers on the other. 

Verdile and colleagues [19] perform a detailed characterization of the intestinal epithelial cells lining the intestinal tract in rainbow trout (*Oncorhynchus mykiss*) throughout the first year of development. The analysis was performed at typical time points of in vivo feeding trials (50, 150, and 500 g) in order to establish accurate reference values, especially across the productive cycle of animals raised in standardized conditions. 

Imperatore and colleagues [20] analyze the effect of 3,5-diiodo-L-thyronine (3,5-T2), an endogenous metabolite of thyroid hormones whose administration to rodents fed a high-fat diet (HFD) prevents body weight increase and reverts the expression pattern of pro-inflammatory factors associated with HFD, in a diet-induced obese (D.I.O.) zebrafish (*Danio rerio*) model. The authors reveal that the effects of 3,5-T2 on fish intestines and brains can deviate from those shown in obese mammals; through the expression of different inflammatory markers, they determined that 3,5-T2 sustained or increased inflammation in the intestine when administered with the obesity-inducing diet.
